# Endothelial Cell mTOR Complex-2 Regulates Sprouting Angiogenesis

**DOI:** 10.1371/journal.pone.0135245

**Published:** 2015-08-21

**Authors:** Maikel A. Farhan, Katia Carmine-Simmen, John D. Lewis, Ronald B. Moore, Allan G. Murray

**Affiliations:** 1 Department of Medicine, University of Alberta, Edmonton, Canada; 2 Department of Oncology, University of Alberta, Edmonton, Canada; 3 Department of Surgery, University of Alberta, Edmonton, Canada; BloodCenter of Wisconsin, UNITED STATES

## Abstract

Tumor neovascularization is targeted by inhibition of vascular endothelial growth factor (VEGF) or the receptor to prevent tumor growth, but drug resistance to angiogenesis inhibition limits clinical efficacy. Inhibition of the phosphoinositide 3 kinase pathway intermediate, mammalian target of rapamycin (mTOR), also inhibits tumor growth and may prevent escape from VEGF receptor inhibitors. mTOR is assembled into two separate multi-molecular complexes, mTORC1 and mTORC2. The direct effect of mTORC2 inhibition on the endothelium and tumor angiogenesis is poorly defined. We used pharmacological inhibitors and RNA interference to determine the function of mTORC2 *versus* Akt1 and mTORC1 in human endothelial cells (EC). Angiogenic sprouting, EC migration, cytoskeleton re-organization, and signaling events regulating matrix adhesion were studied. Sustained inactivation of mTORC1 activity up-regulated mTORC2-dependent Akt1 activation. In turn, ECs exposed to mTORC1-inhibition were resistant to apoptosis and hyper-responsive to renal cell carcinoma (RCC)-stimulated angiogenesis after relief of the inhibition. Conversely, mTORC1/2 dual inhibition or selective mTORC2 inactivation inhibited angiogenesis in response to RCC cells and VEGF. mTORC2-inactivation decreased EC migration more than Akt1- or mTORC1-inactivation. Mechanistically, mTORC2 inactivation robustly suppressed VEGF-stimulated EC actin polymerization, and inhibited focal adhesion formation and activation of focal adhesion kinase, independent of Akt1. Endothelial mTORC2 regulates angiogenesis, in part by regulation of EC focal adhesion kinase activity, matrix adhesion, and cytoskeletal remodeling, independent of Akt/mTORC1.

## Introduction

Drug therapy to inhibit tumor neovascularization is used clinically as an adjuvant in chemotherapy–resistant cancers, including renal cell carcinoma, recurrent glioblastoma, and bowel cancer. The rapalog mammalian target of rapamycin (mTOR) inhibitors are used after failure of pro-angiogenic growth factor–receptor tyrosine kinase inhibitors, and in some cases as first line therapy [1]. Rapalog mTOR inhibition decreases Vascular Endothelial Growth Factor (VEGF) production by the tumor to reduce tumor neovascularization and inhibit tumor growth [2,3]. However, this therapeutic approach is limited by the development of resistance of the tumor and microvasculature to the effect of rapalog mTOR inhibition [4,5]. This “escape” of the vasculature from the effects of current mTOR inhibitors emphasizes the need for new agents with durable effects.

In mammalian cells, mTOR is assembled in two distinct signaling complexes: mTOR complex-1 (mTORC1), sensitive to inhibition by rapalog drugs, and mTOR complex-2 (mTORC2) [6]. In addition to the mTOR catalytic subunit, mTORC1 consists of raptor (regulatory associated protein of mTOR), mLST8 (also termed G-protein β-subunit-like protein, GβL, a yeast homolog of LST8), and PRAS40 (proline-rich Akt substrate 40 kDa). mTORC1 activity is best characterized by phosphorylation of ribosomal protein S6 kinase (S6K) and eukaryotic translation initiation factor 4E-binding protein 1 to regulate translation [7]. mTORC2 similarly includes mTOR and mLST8, but raptor is replaced by two mTORC2-specific proteins: rictor (rapamycin-insensitive companion of mTOR), and mSin1 (mammalian stress-activated protein kinase-interacting protein 1). The principal known target of mTORC2 is Akt, a key survival enzyme, and upstream regulator of mTORC1 [7]. The targets of mTORC1 are well-defined, but much less is known regarding mTORC2-mediated effects independent of Akt/ mTORC1.

Pro-angiogenic cues are recognized by activation of several growth factor receptors displayed on the vascular endothelium, and the diverse signals are integrated to recruit key signal transduction pathways in the endothelial cell (EC). For example, the principal endothelial VEGF receptor, VEGF-receptor 2, is coupled to phosphatidylinositide 3 (PI3)-kinase, signaling to the downstream mTOR kinase [8]. In pre-clinical models, mTORC1 inhibition reduces early vessel growth to VEGF stimulation [2,3,9]. Nevertheless, vessel development and tumor growth proceeds in humans treated with rapalog drugs, prompting the investigation of agents that inhibit mTOR in both complexes [10].

The effect of disrupted signaling of the mTORC2 branch point on the PI3 kinase pathway in the endothelium is poorly understood, but may contribute anti-angiogenic effects [11]. In this paper we report that genetic inactivation of mTORC1 activity or inhibition by rapamycin paradoxically upregulates mTORC2 and Akt activity in primary human ECs. Pharmacologic inhibition or genetic disruption of mTORC2 by rictor knock-down optimally blocks VEGF-stimulated angiogenic sprouting of human ECs *in vitro*. Mechanistically, we identify that mTORC2 activity in ECs is needed for cell migration, development of mature matrix adhesion structures, and specifically regulates VEGF-stimulated Src and focal adhesion kinase activity.

## Materials and Methods

### Reagents

Medium 199 (M199), Hank's Balanced Salt Solution (HBSS), fetal bovine serum (FBS), and endothelial cell growth supplement were purchased from Invitrogen (Burlington, ON). VEGF-A was from Peprotech (Princeton, NJ). Rapamycin, PP242, a highly specific mTOR active-site inhibitor [12], and anti-tubulin-α was from Millipore (Temecula, CA). Ku-0063794, a second specific mTOR inhibitor [13] was from Sigma (St. Louis, MO). Anti-Akt1 was from Protein Tech (Chicago, IL). Hiperfect, non-silencing short interfering RNA (siRNA) and Akt1 silencing siRNA were from Qiagen Inc (Mississauga, ON). Human tumor necrosis factor-α (TNFα) was from Cedarlane (Mississauga, ON). Cycloheximide, phalloidin-FITC, anti-vinculin, and DAPI were from Sigma. Anti-S6K was from Abcam (Cambridge, UK). Anti-phospho-Akt^S473^, anti-phospho-S6K^T389^, anti-FAK, anti-phospho-FAK^Y397^, anti-raptor, anti-rictor, and rictor siRNA were from Cell Signaling Technology (Danvers, MA). ON-TARGETplus human raptor siRNA-SMARTpool was from Thermo Scientific (Waltham, MA).

### Cell culture

Human umbilical vein ECs (HUVECs) were isolated as described previously [14]. The protocol for HUVEC isolation was approved by the Research ethics Board of the University of Alberta. The human microvascular endothelial cell line (HMEC-1; ATCC, Manassas, VA). To facilitate study of VEGF as the only proangiogenic factor, cells were washed with M199 twice and incubated in M199 with 10% FBS and 20 ng/mL VEGF for 18 hours before performing the experiments. To optimize VEGF-induced signals, HUVECs were starved in M199 + 1% FBS overnight, then stimulated with 20 ng/ml VEGF.

### RNA interference

HUVECs were seeded at 70–80% confluency and transfected serially twice over two days with either 50 nM non-silencing (siNS) or specific siRNA using Hiperfect transfection reagent according to the manufacturer’s protocol. Protein expression was evaluated by Western blot.

### Western blot

HUVEC monolayers were washed once with ice cold phosphate buffered solution (PBS) and then lysed immediately on ice by RIPA buffer (10 mM Tris, pH 7.4, 100 mM NaCl, 1 mM EDTA, 1 mM EGTA, 5 mM NaF, 2 mM Na3VO4, 0.1% SDS, 0.5% Na deoxycholate, 1% Triton X-100, 10% glycerol, 1 mM PMSF) followed by boiling at 95°C for 5 minutes. The lysates were resolved by SDS-PAGE, blotted on nitrocellulose membranes (Biorad), then immunostained overnight at 4°C in blocking buffer (5% BSA/TBS-Tween20). Proteins were detected using Luminata forte (EMD Millipore, Billerica, MA) and a Fluorchem FC2 CCD camera (Alpha Innotech). Protein bands were equally contrast enhanced by Adobe Photoshop CS3 then quantified by ImageJ.

### Angiogenesis

A 3D angiogenesis assay *in vitro* was done as previously described [15]. Briefly, HUVECs were transfected with siNS or siRictor and were labeled with CellTracker Green (Life Technologies). Cytodex beads were coated with HUVECs (~400 cells/bead) and cultured for 4 hours in (M199, 10%FBS, 20ng/ml VEGF). The beads were washed twice, suspended in fibrinogen (2 mg/mL) containing aprotinin (0.15 U/mL), and 0.625 U/mL thrombin was added. Angiogenesis growth media (M199, 10% FBS, 50 ng/ml VEGF) was then added on top. To inhibit mTORC1 *versus* mTORC1/2, rapamycin (5 nM) or PP242 (1–10 μM) were added, respectively, to both the fibrin gel and the growth media. To study tumor angiogenesis *in vitro*, HUVECs were pre-treated with PP242 (1 uM), ku-0063794 (50 nM) or rapamycin (5 nM) for 24 hours, then labeled with CellTracker Green (Life Technologies). Caki-1 human renal cell carcinoma cells (ATCC; Manassas, VA) were labeled with CellTracker Red. Cytodex beads were coated with HUVECs or Caki-1 (~400 cells/bead) then embedded in fibrin gel. Growth media (M199, 8% FBS) containing Caki-1 (20,000/well) in suspension was then added on top. At least 90 beads per treatment from each experiment were imaged after 18–20 hours incubation in 5% CO2 at 37°C, using a 20X objective lens and a CCD camera equipped inverted fluorescence microscope (Leica, Concord, ON). Scoring was done using OpenLab (PerkinElmer; Waltham, MA).

Assay of angiogenesis *in vivo* was performed as described previously [16]. Briefly, collagen onplants were generated by superimposing two square-gridded nylon meshes on which 30 μl of 4.73 mg/ml rat tail collagen with VEGF (100 ng/onplant) was placed. Following collagen polymerization, the onplants were placed on the chorio-allantoic membrane (CAM) of 10-day-old shell-less chick embryos. Embryos were incubated for 64 hours at 37°C, then the extent of onplant vascularization was quantified. Newly formed vessels were identified by imaging the upper mesh of the onplant with a dissecting microscope. Images were captured at 6.3x using a StereoLumar V12 fluorescence dissection microscope (Carl Zeiss). The angiogenic index of each onplant was determined as the percentage of grids with newly formed blood vessels out of the total number of grids in the upper mesh.

### Electrical cell-substrate impedance sensing (*ECIS)*

Cell-matrix adhesion and cell migration were tested using the ECIS device (Applied BioPhysics Inc., Troy, NY). HUVECs were treated with PP242, siAkt1, siRictor, or siRaptor as indicated. Equal numbers of cells were seeded into gelatin-coated ECIS plates with VEGF (20 ng/ml). Adhesion and migration were then assessed by continuous resistance measurements in optimum growth conditions over 12 hours.

### Apoptosis assay

Apoptosis was measured in HUVECs in optimum growth conditions and after inducing apoptosis. To induce apoptosis, EC monolayers were washed, then incubated with cycloheximide (CHX; 3 μg/mL) and tumor necrosis factor-α (TNFα; 10 ng/mL) for 4 hours. HUVECs were incubated with the FITC-conjugated active caspase-3 reporter, DEVD-FMK, for 30 minutes at 37°C as directed by the manufacturer (Promokine, Heidelberg, Germany). Cells were trypsinized and combined with the floating cells in the medium. After brief washes, cells were analyzed by flow cytometry (LSR-Fortessa).

### Immunofluorescence microscopy

HUVECs were cultured on gelatin-coated glass bottom microwell dishes (MatTek Corp., Ashland, MA). Cells were fixed for 10 minutes in 3% fresh paraformaldehyde in PBS, washed with PBS, then permeabilized and blocked in 3% BSA + 0.1% Triton X-100 in PBS for 30 minutes. Cells were washed in PBS then incubated with phalloidin-FITC or anti-vinculin-FITC, and DAPI in 1% BSA for 1 hour in room temperature. The cells were washed 3 times with PBS. Filamentous actin and vinculin were visualized at 40X using an inverted fluorescence microscope, and focal adhesions were enumerated as described [17].

### G-actin/F-actin in vivo assay

The separation of filamentous actin (F-actin) and globular actin (G-actin) was done according to the manufacturer’s instruction (Cytoskeleton, Denver, CO, USA). The ratios of F-actin to G-actin in ECs were estimated by Western blot.

### Statistical analyses

Data are shown as mean ± SEM. Statistical analysis was performed by 1-way ANOVA as appropriate followed by the Bonferroni post-hoc test. Pairwise comparisons were done by paired Student *t*-test using Prism 5 (Graphpad, San Diego, CA). *P* values <0.05 were considered significant. Each experiment was done at least three times.

## Results

### Exposure to rapamycin activates endothelial Akt

**Fig 1:**
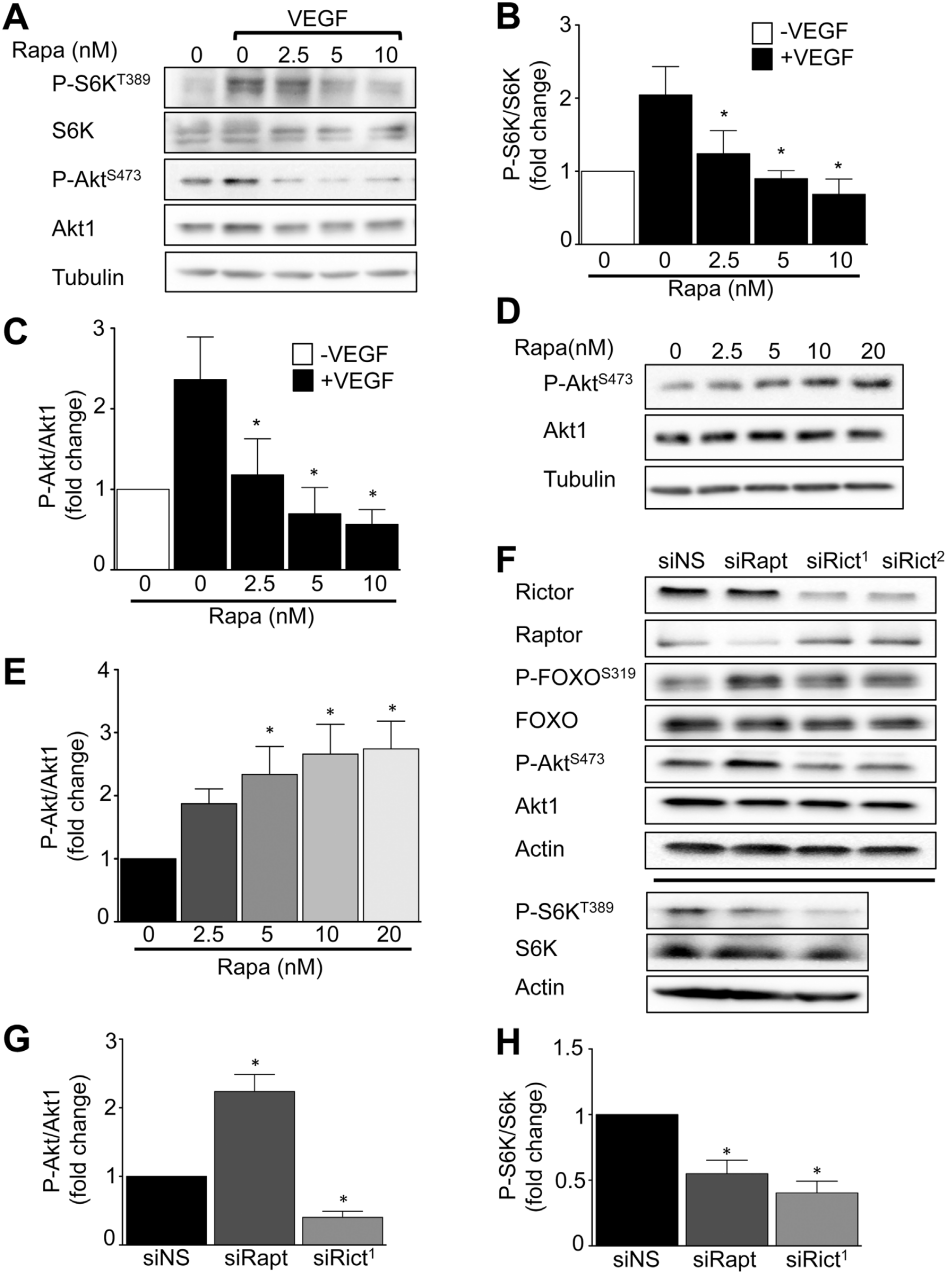
Sustained mTORC1, but not mTORC2, inhibition activates Akt. HUVECs were treated by rapamycin, two different small interfering RNAs against rictor (siRict^1^ and siRict^2^) or raptor (siRapt), or non-silencing siRNA (siNS), then stimulated with 20 ng/mL VEGF as indicated. Akt phosphorylation and S6K phosphorylation were evaluated as described in Materials and Methods. **A**) A representative Western blot of the effect of Rapamycin pretreatment for 1 hour on VEGF-stimulated Akt and S6 kinase phosphorylation in HUVECs. **B)** Quantitation of phospho-S6K. **C)** Quantitation of phospho-Akt (n = 5 independent experiments, *P<0.05 by ANOVA). The effect of rapamycin treatment of HUVEC for 24 hours. **D)** A representative Western blot of HUVEC phospho-Akt, total-Akt1 and tubulin over a range of concentration of rapamycin exposure. **E)** Quantitation of phospho-Akt (n = 5 independent experiments, *P<0.05 by ANOVA). The effect of mTORC1 *versus* mTORC2 disruption on Akt signaling in EC. **F)** A representative Western blot of HUVEC rictor, raptor, phospho-Forkhead box protein O1/3 (P-FOXO1/3), phospho-Akt, total Akt1, phospho-S6K, total S6K, and actin after treatment with siRapt, siRict or siNS. **G)** Quantitation of phospho-Akt. **H)** Quantitation of phospho-S6K (n = 3 independent experiments, *P<0.05 by ANOVA).

In tumor cells, active mTORC1 participates in a negative regulatory loop that inhibits serine 473 (S473) phosphorylation and full activation of Akt. Inhibition of mTORC1 relieves this inhibition, but has a variable and cell-type specific effect on upstream growth factor receptor signaling [18,19]. We sought to determine if this mechanism is present in primary human ECs, since these events could contribute to resistance to rapalog anti-angiogenesis treatments. First, we treated ECs with rapamycin for 1 hour, then stimulated the cells with VEGF. Rapamycin inhibited VEGF-stimulated, mTORC1-dependent phosphorylation of S6K in dose-dependent manner (Fig 1A–1C). We determined rapamycin 5 nM optimally inhibited VEGF-stimulated mTORC1 signaling. Next, we sought to model chronic *in vivo* exposure of ECs to rapamycin by treatment with 5 nM rapamycin for 24 hours. Paradoxically, sustained exposure to rapamycin increased Akt S473 phosphorylation, the mTORC2-dependent site, in a dose-dependent manner (Fig 1D and 1E). Rapamycin treatment at 5 and 10 nM increased Akt S473 phosphorylation ~2.4 ± 0.4 and ~2.7 ± 0.4 fold (mean ± SEM) compared to the control carrier treatment, respectively (Fig 1E). Similarly, sustained inhibition of mTORC1 with rapamycin in human microvascular ECs (HMEC-1) increased Akt S473 phosphorylation (S1 Fig).

To determine if mTORC2- has the same effect as mTORC1- inactivation, we specifically disrupted EC mTORC2 or mTORC1 using siRNA targeted against rictor or raptor, respectively [20,21]. We observed that sustained mTORC1 disruption persistently blocked S6K phosphorylation, but increased phosphorylation of Akt S473 ~2.5 fold and its down stream target, FOXO1/3 S319, compared to control (Fig 1F and 1G). Conversely, mTORC2 disruption markedly lowered Akt S473 phosphorylation compared to control (Fig 1G). Notably, specific disruption of mTORC2 also blocked FOXO and S6 kinase phosphorylation (Fig 1F and 1H). These data indicate sustained inactivation of mTORC1, but not mTORC2, relieves feedback inhibition to drive hyper-activation of the PI3 kinase/ Akt pathway in primary human ECs.

Next, we sought to study the effect of dual mTORC1 and mTORC2 inhibition on VEGF-stimulated EC Akt activation. Selective mTOR kinase inhibitors are active against mTOR in both complexes [12]. Pretreatment of EC with PP242 or Ku-0063794 inhibited VEGF-stimulated Akt activation in a dose-dependent manner, and we determined the optimal inhibitory concentration of the compounds (data not shown). Like rictor knock-down to disrupt mTORC2 formation (Fig 1F–1H), sustained pharmacologic inhibition of mTORC1 + mTORC2 using either PP242 or Ku-0063794 did not increase Akt phosphorylation or Akt activity in EC (S1 and S2 Figs).

### mTORC1 sustained inhibition increases resistance to apoptosis and pre-sensitized EC to angiogenic cues

**Fig 2:**
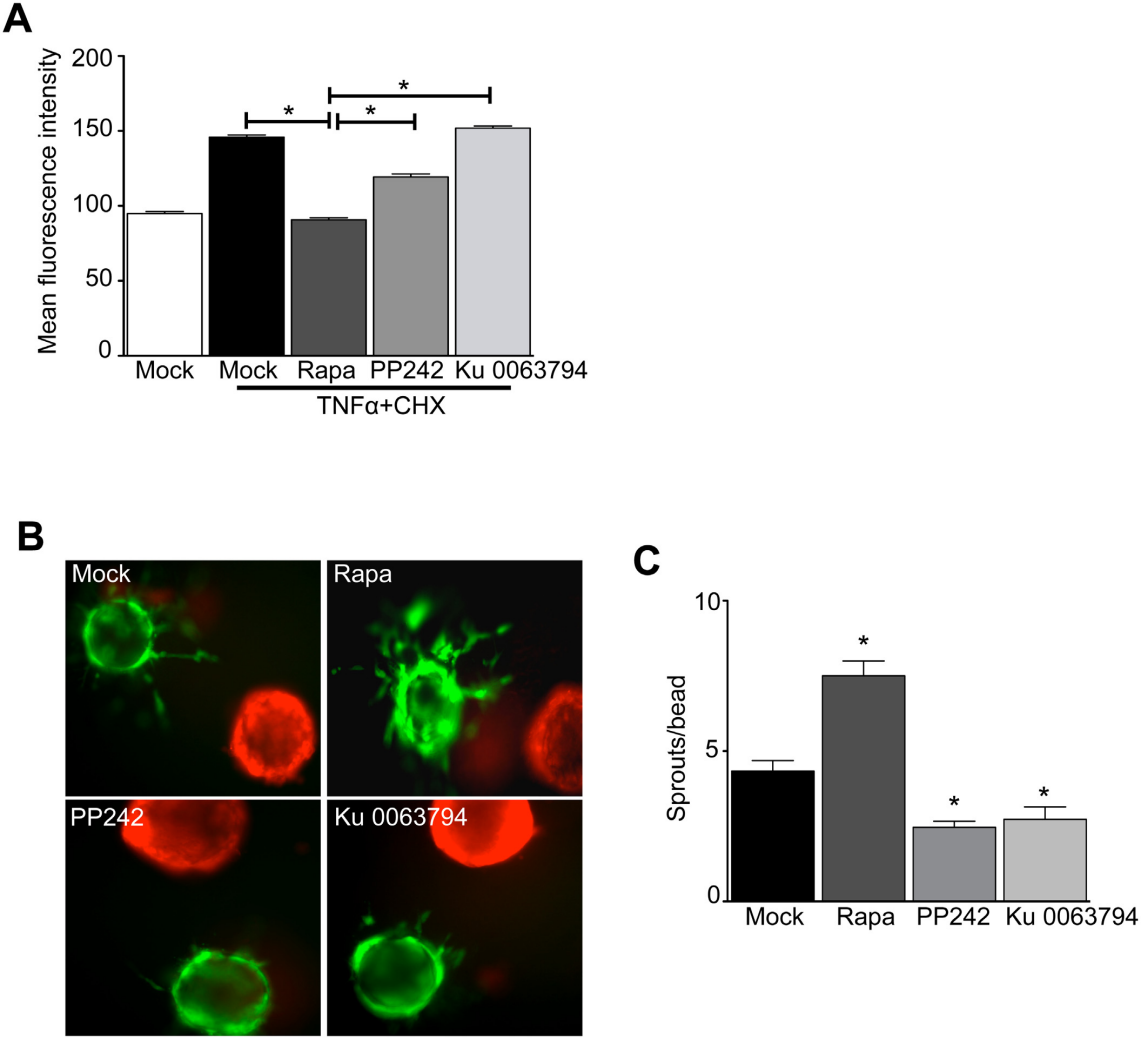
mTORC1 inhibition primes endothelial cells to resist apoptosis and to respond to tumour-derived pro-angiogenic cues. HUVECs were treated with Rapamycin (5 nM), PP242 (1 uM), or Ku 0063794 (50 nM) for 24 hours. **A)** The EC were challenged with tumor necrosis factor-α (TNFα) + cycloheximide (CHX) for 4 hours in normal growth conditions. Active cleaved caspase-3 was detected by DEVD-FMK-FITC and analyzed by flow cytometry as described in Methods. Quantitation of active caspase-3 in mock-, rapamycin-, PP242-, and Ku 0063794-treated ECs (n = 4 independent experiments, *P<0.05 by ANOVA). **B)** Evaluation of EC angiogenic sprouting to tumor-derived growth factors *in vitro*. HUVEC were pretreated with Rapamycin (5 nM), PP242 (1 uM), or Ku 0063794 (50 nM) for 24 hours, then mounted on Cytodex beads (green) and were embedded with renal cell carcinoma cell-coated beads (red) in 3D fibrin gels as described in Methods, then co-cultured without additional growth factor supplementation or inhibitors. Representative images of EC sprouts after 18 hours incubation. **C)** Quantitation of the number of sprouts per bead (n = 3 independent experiments, *P<0.05 by ANOVA).

We evaluated the effect of sustained rapalog exposure on EC function. Akt activity mediates an important cellular pro-survival pathway, hence we sought to determine the effect of extended mTORC1/2 *versus* mTORC1 inactivation on EC apoptosis. We challenged the EC with pro-apoptotic factors, then quantified active cleaved caspase 3 expression among rapamycin-, PP242-, or Ku-0063794-pretreated ECs. We observed that extended mTORC1 inhibition increased EC resistance to apoptosis *versus* mock-treated, or mTORC1/2 inhibited EC (Fig 2A). Conversely, treatment with the PP242 or Ku-0063794 compound did not confer similar resistance. Further, we investigated the effect of extended mTORC1 inhibition on sprouting angiogenesis of human ECs to tumor cells. ECs were pre-treated with rapamycin, PP242 or Ku-0063794 for 24 hours, then embedded in a 3D fibrin matrix with human RCC cells in the absence of exogenous pro-angiogenic growth factors and compounds. Tumor cells stimulated EC sprouting, which was increased by rapamycin but not PP242 or Ku 0063794 pre-exposure (Fig 2B and 2C). Similarly, rapamycin pre-exposure increased EC migration toward tumor cells in Boyden chamber chemotaxis assay (data not shown). These observations suggest that mTORC1/2 inactivation may be more effective than mTORC1 inhibition to block tumor neoangiogenesis.

### mTORC1 + mTORC2 dual inhibition reduces VEGF-induced angiogenesis

**Fig 3:**
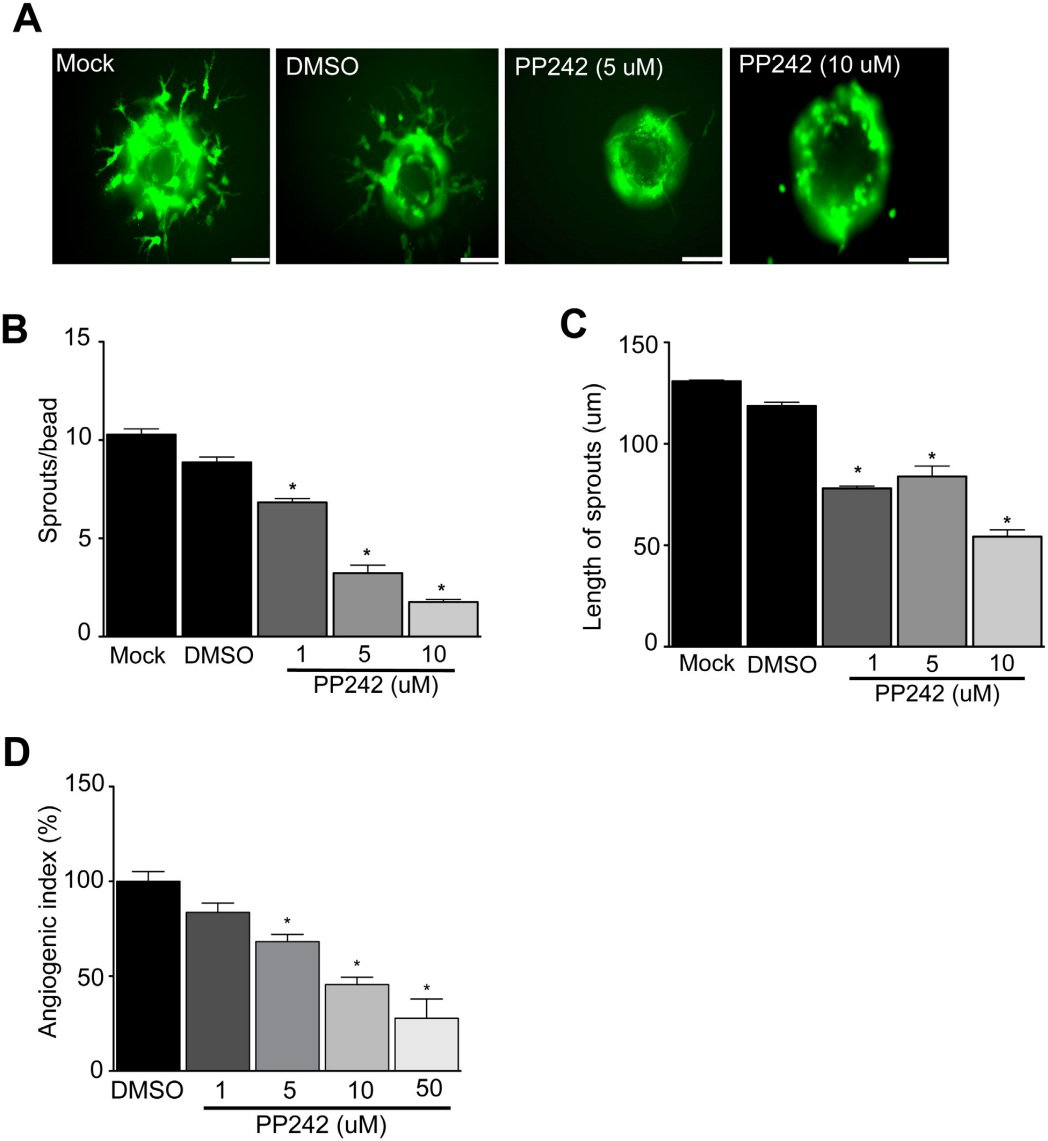
mTORC1/2 dual inhibition blocks VEGF-mediated angiogenesis. HUVEC-coated Cytodex beads were embedded in fibrin gels as in Fig 2, then the EC were stimulated with 50 ng/ml VEGF, and were treated with PP242 or carrier as indicated. **A)** Representative images of EC sprouts after 18 hours incubation. **B)** Quantitation of the number of sprouts per bead. **C)** Quantitation of the length of the sprouts (n = 3 independent experiments, *P<0.05 by ANOVA, scale bar = 95 um). **D)** Collagen gel onplants containing VEGF (100 ng/onplant) and PP242 or carrier, were placed on chicken embryo CAM as described in Methods. Quantitation of neovascularization after 64 hours of exposure to VEGF supplemented with 1, 5, 10 or 50 uM PP242 (n > 48 onplants or 16 chicken embryos per group, *P<0.05 by ANOVA).

Next we studied the effect of mTOR inhibition on sprouting angiogenesis of human primary ECs in response to VEGF. Primary human ECs were loaded on microcarrier beads and embedded in 3D fibrin gels, then treated with PP242. Consistent with the effect on tumor angiogenesis, PP242 treatment markedly reduced human EC sprout formation, and reduced the elongation of the few endothelial sprouts that developed in response to VEGF (Fig 3A, 3B and 3C). Similar findings were obtained using a second mTORC1/2 inhibitor in microvascular ECs (S3 Fig).

To determine the effect of mTORC1/2 inhibition on VEGF-stimulated neovascularization *in vivo*, while avoiding confounding effects of the compound on stromal cell production of VEGF, PP242 or carrier was added to VEGF-loaded onplants deposited on the chick embryo CAM, then microvessel density was measured after 3 days. As shown in (Fig 3D) and (S4 Fig), we observed that PP242 inhibited new vessel formation in a concentration-dependent manner. It is worth noting that the inhibitor concentration required to inhibit neovascularization was higher *in vivo* than *in vitro* likely due to the diffusion-associated loss of PP242 concentration during the CAM assay. Taken together, these data indicate that dual inhibition of mTORC1/2 activity has potent anti-angiogenesis effects, acting directly on the vascular EC.

### Specific mTORC2 disruption inhibits VEGF-stimulated angiogenesis

**Fig 4:**
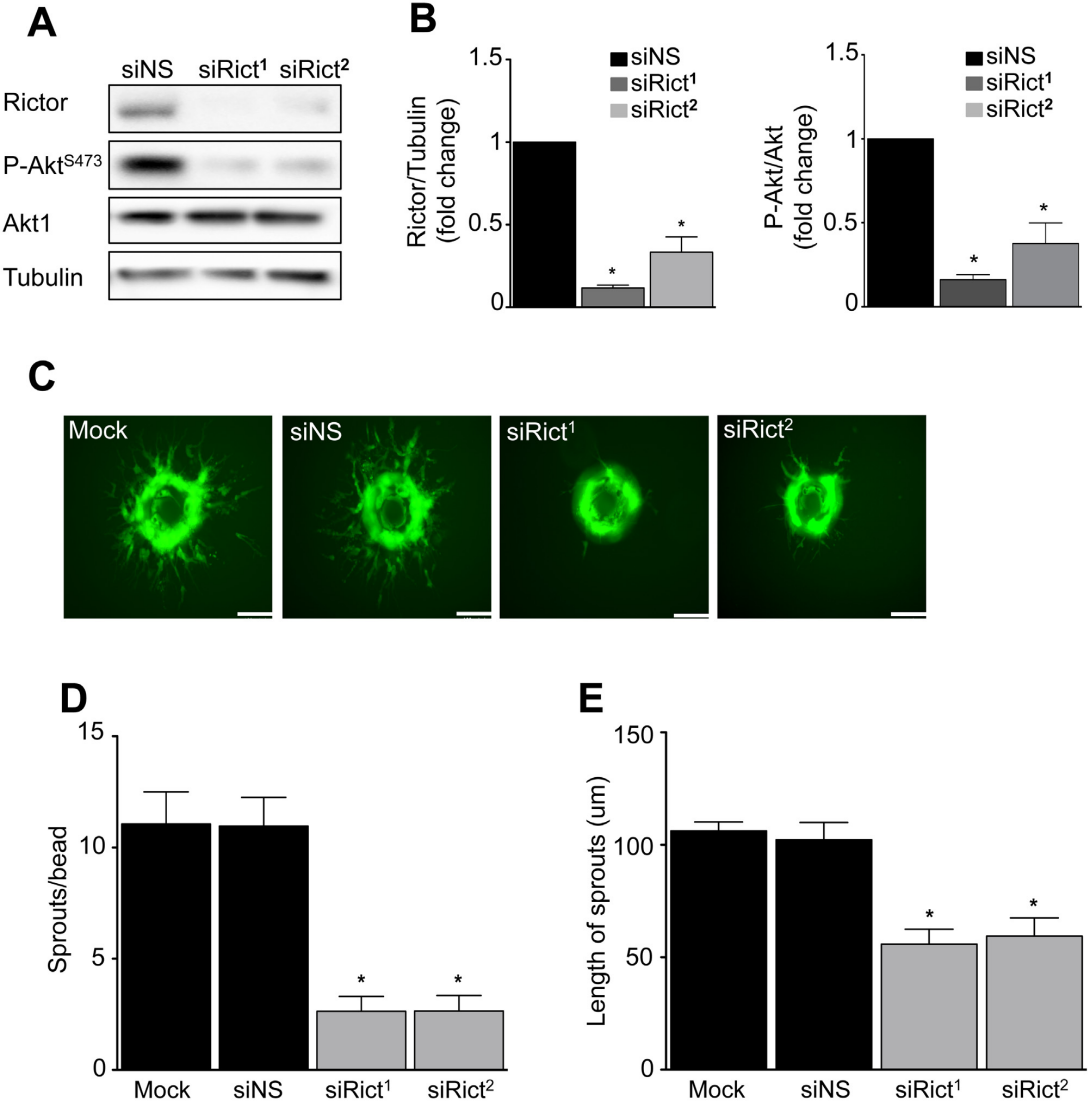
mTORC2 inactivation inhibits VEGF-mediated angiogenesis. HUVECs were transfected with either of two small interfering RNAs (siRNAs) targeting rictor (siRict^1^ and siRict^2^) or a non-silencing siRNA (siNS) control. **A)** Representative Western blot of the effect of rictor knockdown on rictor, and phospho-Akt. **B)** Quantitation of rictor and phospho-Akt (n = 3 independent experiments, *P<0.05 by ANOVA). **C)** The effect of rictor knockdown on angiogenic sprouting *in vitro*. HUVEC were transfected with siRict^1^, siRict^2^, or siNS, then mounted on Cytodex beads, embedded in a fibrin gel, and stimulated with 50 ng/ml VEGF as in Fig 3. Representative images of EC sprouts after 18 hours incubation. **D)** Quantitation of the number of sprouts per bead. **E)** Quantitation of the length of the sprouts (n = 3 independent experiments, *P<0.05 by ANOVA, scale bar = 95 um).

In complementary experiments, we used RNA interference to specifically inactivate mTORC2 activity in ECs. We used two different siRNA against rictor (siRict^1^ and siRict^2^) to disrupt mTORC2, and confirmed the effect of the knock-down in EC by Western blot of rictor expression, and mTORC2 activity by Akt S473 phosphorylation (Fig 4A). Rictor siRNA treatment reduced EC rictor and Akt S473 phosphorylation by ~80% (Fig 4B). Specific disruption of mTORC2 markedly inhibited VEGF-stimulated angiogenic sprouting of ECs. Primary human ECs were transfected with siRict, then evaluated for sprouting into 3D fibrin gels. We observed mTORC2 disruption reduced the number of sprouts by ~75% (Fig 4C and 4D). In addition, among the sprouts that developed, mTORC2 disruption markedly reduced EC sprout extension into the fibrin gel (Fig 4E). This indicates that mTORC2 activity is required for angiogenic sprouting, and inactivation of mTORC2 additionally reduces sprout length.

### mTORC2 regulates EC migration and cell-matrix adhesion independent of Akt and mTORC1 activity

Next we sought to determine if Akt-dependent signaling mediates the contribution of mTORC2 to angiogenesis. Akt activity is regulated in parallel by PDK1-dependent T308 phosphorylation in the activation loop of Akt, and enhanced by S473 phosphorylation mediated by mTORC2 to regulate a subset of Akt-dependent responses [22]. Using RNA interference we selectively knocked-down expression of Akt1 (the dominant Akt isoform in ECs) or rictor, then we studied the effect of EC Akt1*versus* mTORC2 loss on ECs *in vitro*. The level of Akt1 after knockdown is shown in (S5 Fig).

**Fig 5:**
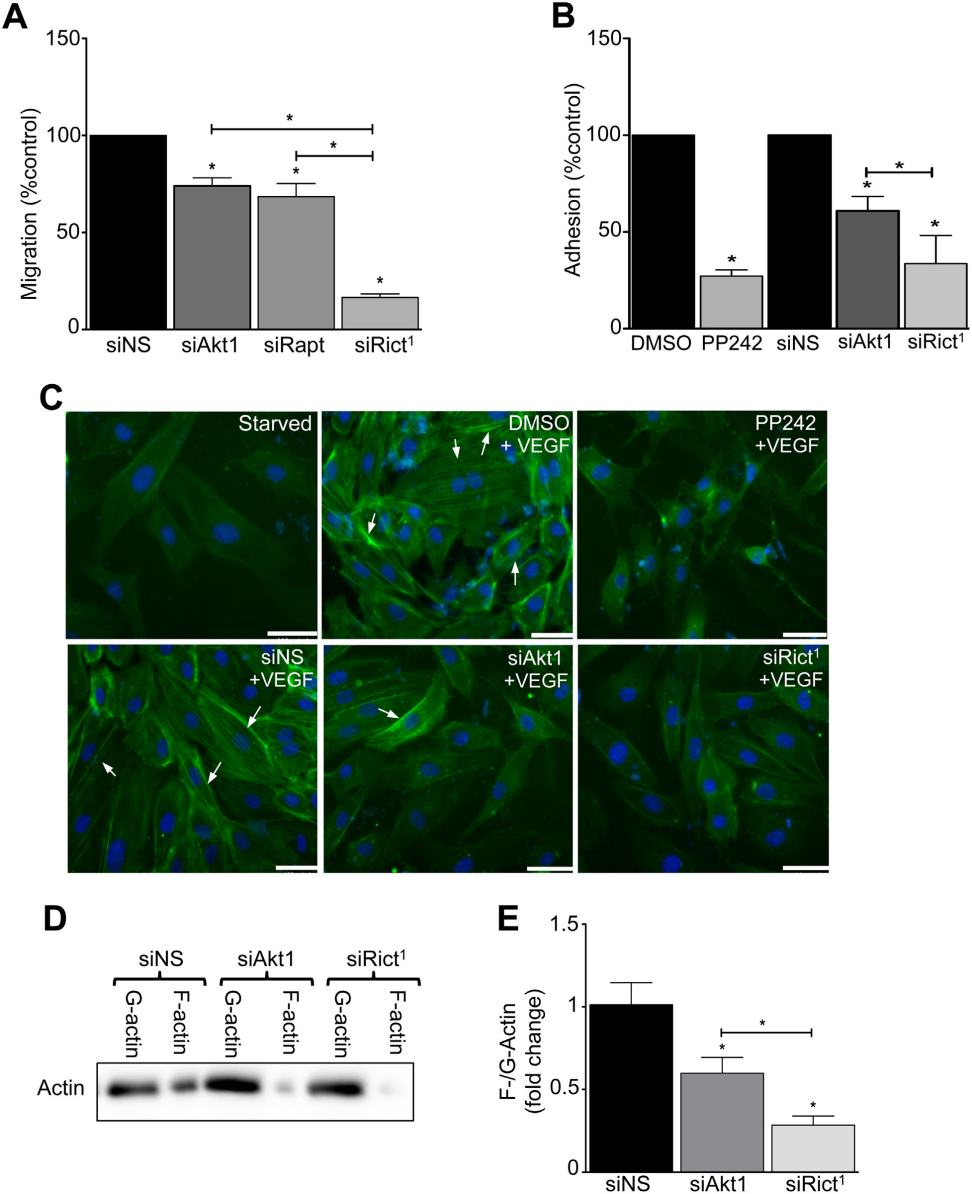
mTORC2 inactivation reduces EC migration, matrix adhesion, and actin polymerization. HUVECs were transfected with siRNA targeting Akt1 (siAkt1), rictor (siRict^1^) or raptor (siRapt). **A)** The ECs were seeded on gelatin-coated electrodes at equal density to reach confluence. The recovery of electric impedance was measured following delivery of a high electric current through an electrode to create a defect in the EC monolayer as described in Methods. Data are represented as the relative rate of migration per hour *versus* the control (n = 3 independent experiments, *P<0.05 by ANOVA). HUVECs were transfected with siRict^1^, or siAkt1, or treated with PP242. **B)** The EC were seeded equally on gelatin-coated electrodes, then adhesion was evaluated using electrical impedance measurements. Data are represented as the relative rate of adhesion *versus* the controls (n = 3 independent experiments, *P<0.05 by ANOVA). **C)** The EC were serum-starved overnight, and stimulated with VEGF for 10 minutes or not (upper left). Fluorescence images of phalloidin-stained filamentous (F)-actin (green) and DNA (blue) are representative of 3 independent experiments illustrating a marked decrease in VEGF-stimulated F-actin among mTORC2-inactivated EC. **D)** HUVECs were transfected with siAkt1, or siRict^1^, or control siNS. The EC were stimulated with VEGF, then globular (G)-actin and F-actin were separated as described in Methods. A representative Western blot illustrates the relative abundance of the F-actin and G-actin in ECs. **E)** Quantitation of the F-/G-actin ratio (n = 5 independent experiments, *P<0.05 by ANOVA).

To examine migration of rictor-, Akt1-, and raptor-deficient ECs, we wounded confluent EC monolayers using a high-field electric current. We observed a modest decrease in the closure of the wound, indicating EC migration was impaired, among both Akt1- and raptor-deficient cells (Fig 5A and S6 Fig). However, loss of rictor conferred a striking decrease in wound closure. EC migration and sprout elongation require EC adhesion and anchorage to the surrounding matrix. Hence we determined the effect of mTORC2-inactivation on cell-matrix interaction. Integrin-mediated cell adhesion was reduced by 73 ± 3% after mTORC1 + mTORC2-inactivation, and by 66 ± 10% (mean ± SEM) among mTORC2-disrupted ECs compared to control ECs, measured by electrical impedance after seeding ECs on gelatin-coated electrodes (Fig 5B and S7 Fig). Notably, we observed disruption or PP242-mediated inhibition of mTORC2 was more potent than inactivation of Akt to block EC adhesion. These results suggest mTORC2 mediates regulation of EC movement and adhesion, independent of Akt1/mTORC1.

In yeast, TORC2 regulates cytoskeleton remodeling independent of Akt, but this is not observed in embryonic fibroblasts isolated from mTORC2-disrupted knockout mice [21,22,23]. Since cytoskeletal remodeling is involved in cell migration and angiogenic sprout elongation, we studied the effect of mTORC2 inactivation on actin polymerization among primary human ECs. Immunofluorescence staining for polymerized actin showed a paucity of stress fibers in VEGF-stimulated, mTORC2-inactivated, adherent ECs compared to control ECs (Fig 5C). Similarly, protein analysis showed that the ratio of filamentous to globular actin was markedly reduced in mTORC2-disrupted adherent ECs (Fig 5D and 5E). Further, the F-/G-actin ratio was significantly reduced in mTORC2-disrupted ECs compared to Akt1-deficient ECs, consistent with mTORC2-mediated, Akt1-independent regulation of actin remodeling.

### mTORC2 regulates focal adhesion kinase

**Fig 6:**
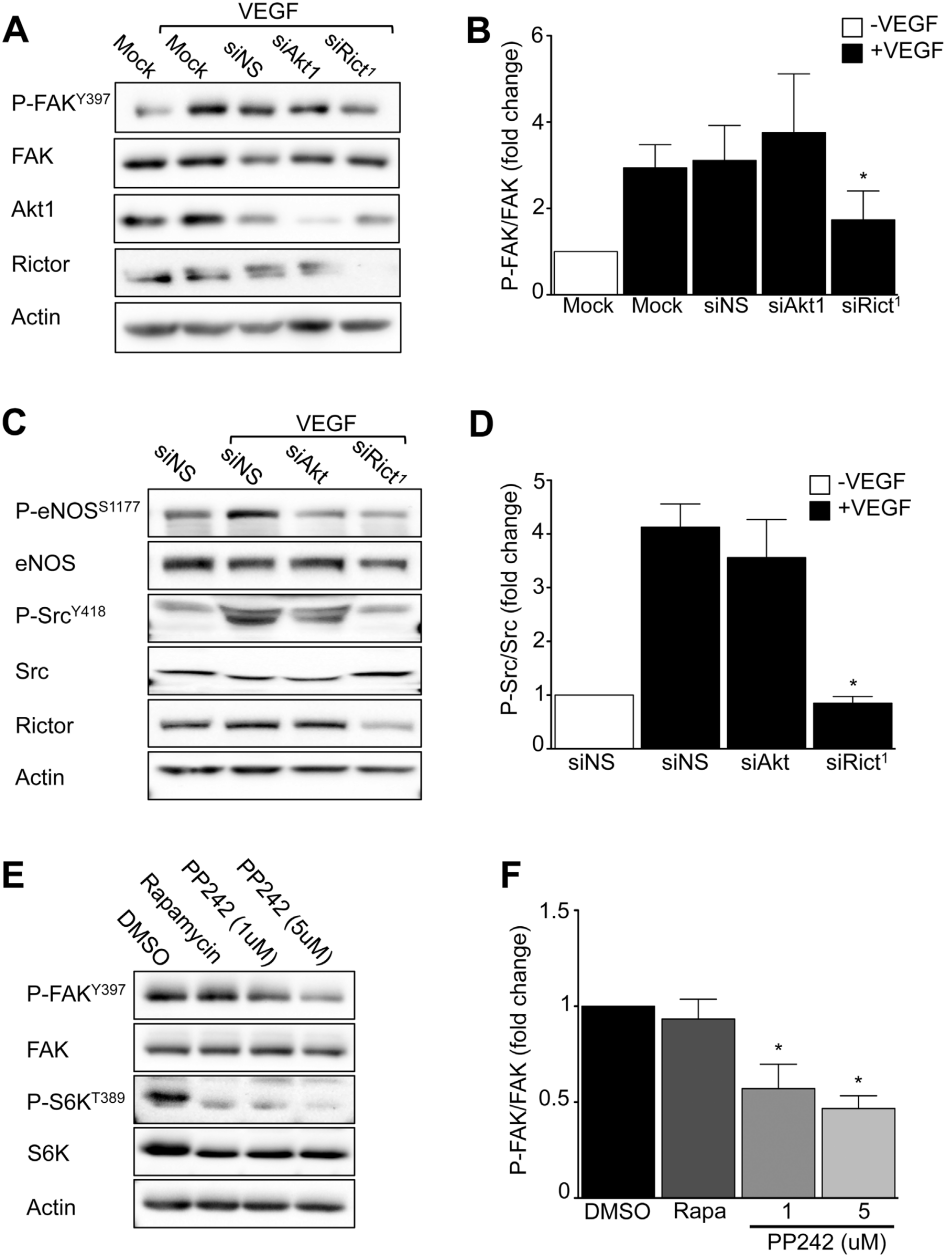
mTORC2 inactivation inhibits focal adhesion kinase activity. HUVECs were transfected with siRict^1^ or siAkt1, then stimulated with 20 ng/mL VEGF for 10 minutes as indicated. **A**) A representative Western blot of EC phospho-focal adhesion kinase (P-FAK), total FAK, total Akt1, total rictor and actin. **B)** Quantitation of P-FAK (n = 4 independent experiments, *P<0.05 by ANOVA). **C)** A representative Western blot of EC phospho-eNOS, and phospho-Src, illustrates that mTORC2 disruption, but not Akt1 inactivation, blocks VEGF-stimulated Src activation (n = 3 independent experiments). Knockdown of either rictor or Akt1 similarly blunts eNOS phosphorylation. **D)** Quantitation of P-Src (n = 3 independent experiments, *P<0.05 by ANOVA). **E)** The effect of sustained mTORC1 or mTORC1/2 inhibition on EC FAK activation. HUVECs were treated with PP242 or rapamycin and stimulated with VEGF overnight. A representative Western blot of EC P-FAK, and P-S6K (n = 3 independent experiments). **F)** Quantitation of P-FAK (n = 3 independent experiments, *P<0.05 by ANOVA).

Defective adhesion, motility, and stress fiber formation in mTORC2-inactivated ECs suggested formation of focal adhesion complex structures that mediate cell interactions with extracellular matrix might be defective. Focal adhesion kinase (FAK) is a principal regulator linking growth factor signals to cell-matrix adhesion and cytoskeleton remodeling [24]. Therefore, we investigated the effect of mTORC2-disruption on FAK activity. VEGF-stimulated FAK Y397 auto-phosphorylation was reduced in mTORC2-disrupted EC monolayers compared to controls, whereas Akt1 knockdown had no effect (Fig 6A and 6B). Moreover, VEGF-stimulated FAK phosphorylation and Src Y418 phosphorylation, an upstream regulator of FAK subcellular localization and activity [25] was completely inhibited in mTORC2- *versus* Akt1-inactivated ECs (Fig 6C and 6D). Similarly, inhibition of mTORC2 for 18 hours dramatically decreased FAK phosphorylation among PP242- (Fig 6E and 6F) or Ku-0063794- (S1 Fig) treated EC, whereas mTORC1 inhibition with rapamycin did not affect FAK activity. In contrast, phosphorylation of eNOS, a known substrate of Akt, was similarly blunted by rictor or Akt1 knockdown in response to VEGF stimulation (Fig 6C).

**Fig 7:**
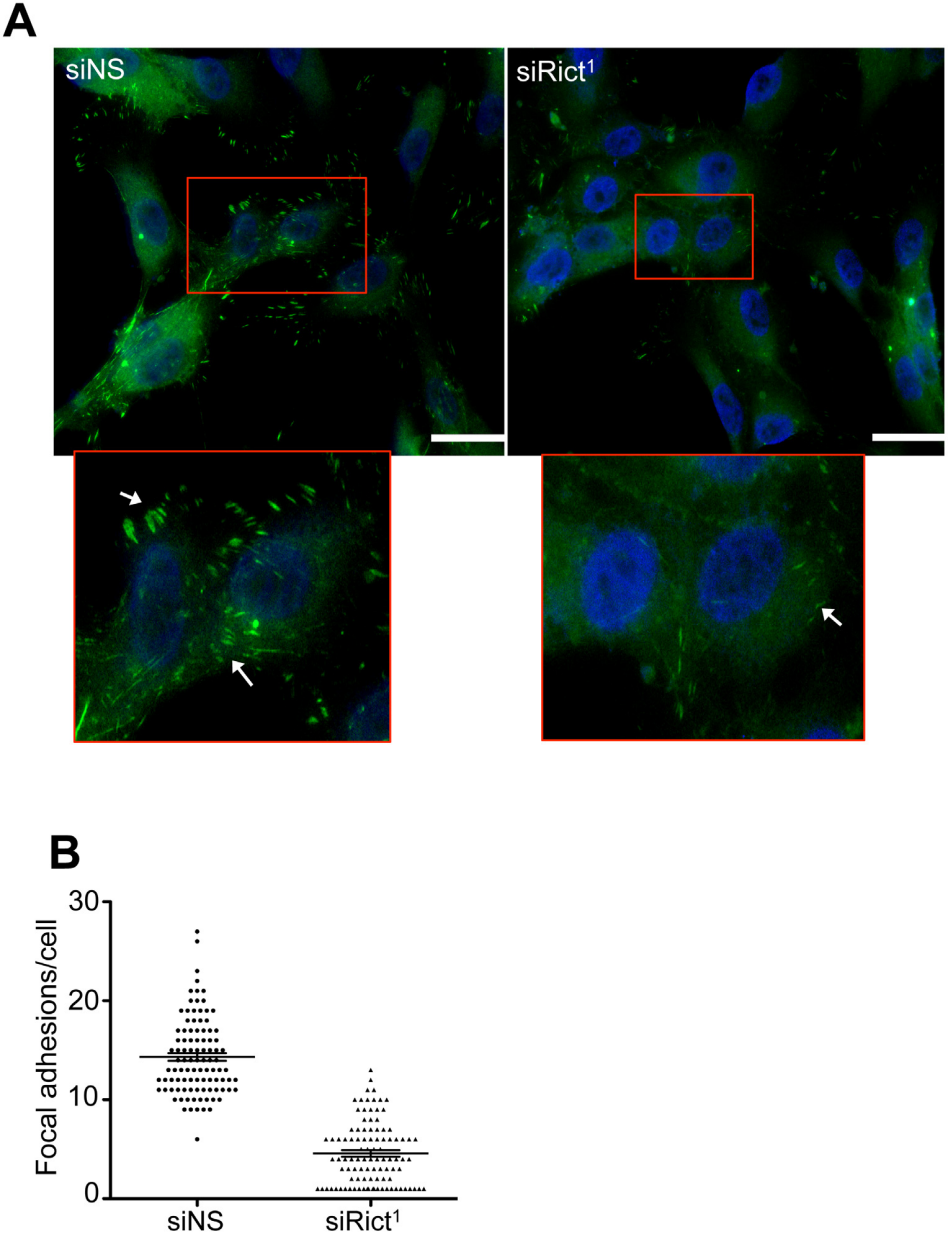
mTORC2 inactivation inhibits focal adhesion formation. HUVECs plated on gelatin matrix were transfected with small interfering RNA against rictor (siRict^1^) or non-silencing siRNA (siNS). **A)** Representative fluorescence image of EC immuno-stained for the formation of vinculin-rich focal adhesions (green) and DNA (blue). **B)** Quantitation of the number of focal adhesions per cell, of 100 ECs pooled from three independent experiments.

Finally, we directly examined the effect of mTORC2 inactivation on matrix adhesion structures in EC. Adherent mTORC2-disrupted ECs were immunostained for the focal adhesion complex protein, vinculin, and the number of complexes was quantitated (Fig 7A and 7B). We observed the number of focal adhesions per EC was reduced in mTORC2-inactivated ECs by 69 ± 2% (mean ± SEM) *versus* control ECs (Fig 7B). Together, these results indicate that mTORC2 regulates FAK activation and EC focal adhesion formation independent of Akt1.

## Discussion

At therapeutic concentrations, rapalog drugs primarily inhibit tumor neovascularization through inhibition of mTORC1-dependent VEGF production [2,3], to blunt tumor angiogenesis [9,26,27]. However, rapalog treatment is limited by escape of the tumor and vasculature from drug inhibition, and subsequent tumor progression [5,28,29]. Indeed withdrawal or interruption of angiogenesis inhibitor treatment may be associated with a flare of angiogenesis and tumor growth [30,31,32,33]. Understanding the events in the endothelial cell underlying the inhibitor drug effects on angiogenesis will guide development of better drugs that target tumor neovascularization. We examined the differential effects on angiogenesis of mTORC2 inactivation or dual mTORC1/2 inhibition *versus* selective mTORC1 inhibition in EC.

We observed that sustained inhibition of mTORC1 with rapamycin paradoxically up-regulated endothelial mTORC2 and Akt activity, promoted resistance of the EC to pro-apoptotic stress conditions, and sensitized EC to cancer cell-stimulated angiogenic sprouting. Conversely, dual inhibition of mTORC1/2 prevented hyper-stimulation of the PI3 kinase pathway, and markedly decreased tumor- and VEGF-mediated angiogenic sprouting among human primary ECs in 3D angiogenesis *in vitro*. Selective gene silencing experiments showed that mTORC2 disruption was sufficient to prevent PI3 kinase pathway hyper-activation in the endothelium, did not confer increased sensitivity to VEGF pro-angiogenic stimulation, and inhibited angiogenesis both *in vitro* and *in vivo* more effectively than mTORC1 inhibition. Moreover, disruption of mTORC2 had an additive effect to Akt1 loss to directly inhibit angiogenic sprouting. We demonstrated that mTORC2 activity regulates EC sprout extension in 3D matrices, which correlates with defects in migration, cell-matrix adhesion, cytoskeleton remodelling, and focal adhesion formation. These effects are linked to Akt1-/mTORC1-independent, but mTORC2-dependent regulation of VEGF-stimulated FAK activation in ECs.

VEGF-receptor recruitment of the PI3 kinase/ Akt/ mTORC1 pathway in the EC plays a critical role in embryonic vascular development, and postnatal neovascularization in several important clinical contexts. Pathological tumor angiogenesis is linked to VEGF-stimulated PI3 kinase/ Akt and mTORC1 activation in the endothelium [2,9,34]. These direct effects of mTORC1 inhibitors on the endothelium likely contribute to an anti-angiogenic effect of rapalog drug adjuvant treatments for advanced cancers. However, escape of the vasculature from the effects of current rapalog mTORC1 inhibitors may by blocked by investigational agents with dual effects to inhibit mTOR activity in both complexes [10,35]. Emerging preclinical studies of mTOR active site inhibitors demonstrate variable control of tumor growth [11,36,37,38,39,40]. However, the relative effects of investigational mTOR active-site inhibitory drugs on tumor cell growth, production of vascular growth factors, *versus* direct effects on the host vasculature in *in vivo* mouse xenograft models are difficult to determine. The current data indicate that the mTOR active site inhibitory agents have direct anti-angiogenic effect on the vascular endothelium.

Recent work has identified complex feedback regulation between mTORC1 inhibition and growth factor-dependent PI3 kinase activity in cancer cells, and cancer cell responses to both receptor tyrosine kinase and androgen receptor stimulation [18,41]. The signal transduction pathway is mediated upstream by PI3 kinase isoform activation by the receptor, then direct and indirect activation of downstream mTORC2, Akt, and mTORC1 to regulate cell metabolism and growth. Inhibition of mTORC1 with rapamycin relieves constitutive inhibition, and further hyper-activates the upstream pathway [18,41]. Our data indicate that a similar EC adaptation to sustained disruption of mTORC1 signalling can prime the endothelium to resist growth factor deprivation, and to respond robustly to tumor-derived pro-angiogenic cues upon interruption in drug administration. Among tumor cells, chronic mTORC2 inhibition may be insufficient to prevent PI3 kinase pathway hyper-stimulation, and hyper-phosphorylation of the Akt target, FOXO1/3 [42]. Our data highlights that a tumor may nevertheless be targeted indirectly through the normal EC mTOR signaling pathway which maintains sensitivity to mTORC2 inhibition.

In addition to the important regulatory effects of mTORC2 on cell metabolism and regulation of growth factor receptor activity mediated through Akt-dependent signaling, we identify a new mTORC2-dependent pathway to regulate cytoskeletal remodelling in angiogenic EC. In yeast, TORC2 regulation of actin structures is recognized, and is shown to be mediated through a PKC homolog [23]. However, mTORC2 regulation of cytoskeleton dynamics in mammalian cells has been controversial. Although disruption of mTORC2 by RNA interference in tumor cell lines affects cell shape, no effect of mTORC2 loss is seen in mouse embryonic fibroblasts derived from either rictor or mLST8-knockout mice [22]. In response to VEGF stimulation, we observe a reduction in angiogenic sprouting of primary human ECs, and a marked decrease in the length of sprout extension after inactivation of mTORC2 in VEGF-stimulated 3D angiogenesis. Defective EC adhesion, migration, and actin remodeling were strikingly more pronounced by mTORC2 inactivation compared to Akt1 knockdown, consistent with an independent contribution of mTORC2 signalling. This correlates with defective formation of organized focal adhesion complexes that anchor actin stress fibres to enable cell contractility and movement.

We identify FAK as a novel downstream effector coupled to VEGF-stimulated mTORC2 activity, independent of Akt/mTORC1. Once EC bind to extracellular matrix, the integrin intracellular domain promotes local assembly of structural and signal transduction molecules, such as Src and FAK, to support actin polymerization and cell migration [43,44]. VEGF stimulation induces remodelling of the complexes associated with Src and FAK phosphorylation. Observations in FAK knockout mice provide direct evidence supporting the role of FAK in angiogenesis, as genetic deletion of FAK in mice is embryonic lethal due to cardiovascular defects [24,45]. Similarly, both rictor and mLST8 subunits of mTORC2 are strongly expressed in the developing vasculature, and mTORC2-disrupted mutant mice die with defective vascular development [22].

The current data suggest mTORC2 indirectly regulates FAK activity, since FAK Y397 phosphorylation is not expected to be mediated by the mTOR serine-threonine kinase activity. The integrin-linked kinase (ILK) has been previously identified both outside and as a component of the mTORC2, and shown to participate in regulation of Akt [46]. Our data indicates that FAK regulation is dependent on mTOR activity, rather than ILK, since FAK phosphorylation is abolished both with disruption of mTORC2 formation after rictor knock-down, and by specific pharmacologic inhibition of mTOR activity. Finally, our data is consistent with previous findings that VEGF-stimulated FAK activation is dependent on VEGFR2 association with integrin, and induction of Src activity in an amplification loop [25,47,48,49]. Our data indicates VEGF-stimulated mTORC2 activity is required upstream of both Src and FAK to remodel matrix adhesion sites.

In summary, our results show that sustained mTORC1 inhibition activates maladaptive PI3 kinase signaling to Akt and responsiveness to proangiogenic growth factors in primary human ECs. In contrast, mTORC2 inactivation prevents Akt and downstream FOXO1/3, or S6 kinase hyper-stimulation. Moreover, mTORC2 regulates EC matrix adhesion, motility, and angiogenesis *in vitro* independent of downstream Akt-regulated events. These data indicate that mTORC2 in the endothelium is an attractive target to inhibit pathologic neoangiogenesis.

## Supporting Information

S1 Fig Sustained mTORC1+2, but not mTORC1, inhibition reduces EC Akt and focal adhesion kinase activation. **A)** HMEC-1were treated with the mTORC1/2 inhibitor Ku 0063794 (50 nM), or Rapamycin, and stimulated with VEGF overnight. A representative Western blot of EC phospho-FAK, total FAK, phospho-Akt, total Akt and actin (n = 3 independent experiments).
(TIF)

S2 Fig Sustained mTORC1+2 inhibition reduces Akt activity. HUVECs were treated with PP242 overnight. A representative Western blot of EC phospho-Akt, phospho-FOXO1/2, phospho-eNOS and actin (n = 3 independent experiments).
(TIF)

S3 Fig mTORC1+2 inhibition reduces VEGF-stimulated sprouting angiogenesis in microvascular EC. HMEC-1 mounted on Cytodex beads were embedded in a fibrin gel as in Fig 3, were treated by Ku 0063794 (50 nM), Rapamycin, or carrier, and stimulated with VEGF for 18 hours. Representative images of angiogenic sprouting are shown in the upper panels. Quantitation of the number of sprouts per bead (lower panel; n = 4 independent experiments, *P<0.05 by ANOVA, scale bar = 95 um).
(TIF)

S4 Fig mTORC1 + 2 inhibition inhibits angiogenesis *in vivo*. Collagen gels containing VEGF 100 ng/mL, or VEGF + PP242 at the indicated concentration, were cultured on chick CAMs as described in Methods. Newly formed vessels growing into the implant (arrows) are identified.
(TIF)

S5 Fig The effect of RNAi targeted to EC Akt1. HUVECs were transfected with non-silencing (siNS) or siRNA against Akt1 (siAkt1). Representative Western blot of EC Akt1 and tubulin.
(TIF)

S6 Fig The effect of rictor, Akt, or raptor loss on endothelial cell migration. HUVEC were transfected with siRNA against rictor, Akt, or raptor, then seeded on gelatin-coated electrodes at high density and grown to confluence as described in Methods. The monolayer was focally disrupted by an electrical pulse. Continuous electrical impedance values are shown. Representative of 3 independent experiments.
(TIF)

S7 Fig The effect of rictor, Akt, or raptor loss on endothelial cell adhesion. HUVEC were transfected with siRNA against rictor, Akt, or raptor, or treated with PP242 as indicated. The EC were then seeded on gelatin- coated electrodes as described in Methods. Continuous electrical impedance values are shown. Representative of 3 independent experiments.
(TIF)
