# Supplementary figures and images for: Endothelial Cell mTOR Complex-2 Regulates Sprouting Angiogenesis
Source: PLoS One. 2015 Aug 21;10(8):e0135245. doi: 10.1371/journal.pone.0135245 (PMC4546419; doi:10.1371/journal.pone.0135245)

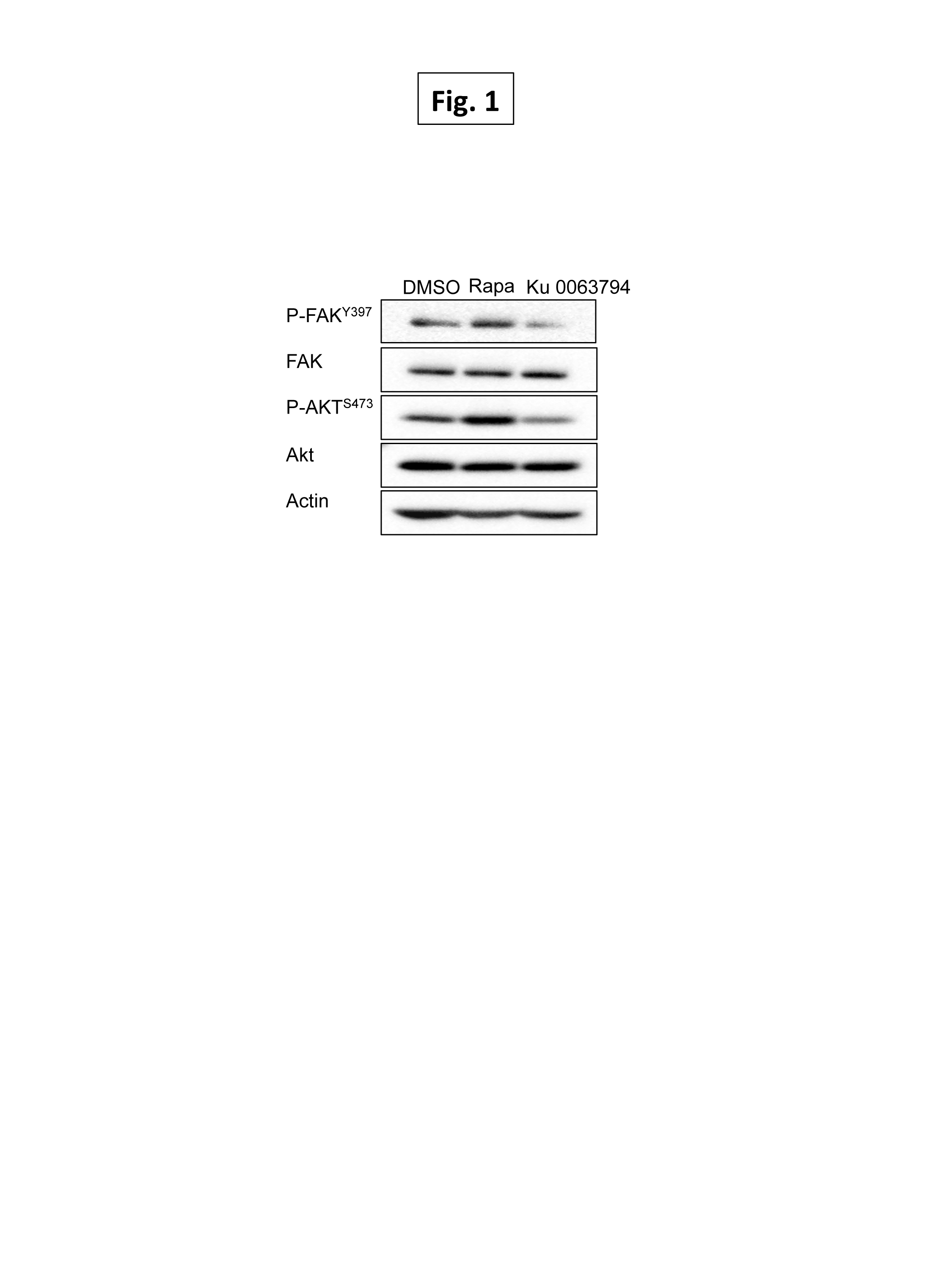

Supplement: S1 Fig — A) HMEC-1were treated with the mTORC1/2 inhibitor Ku 0063794 (50 nM), or Rapamycin, and stimulated with VEGF overnight. A representative Western blot of EC phospho-FAK, total FAK, phospho-Akt, total Akt and actin (n = 3 independent experiments). (TIF) [file pone.0135245.s001.tif]

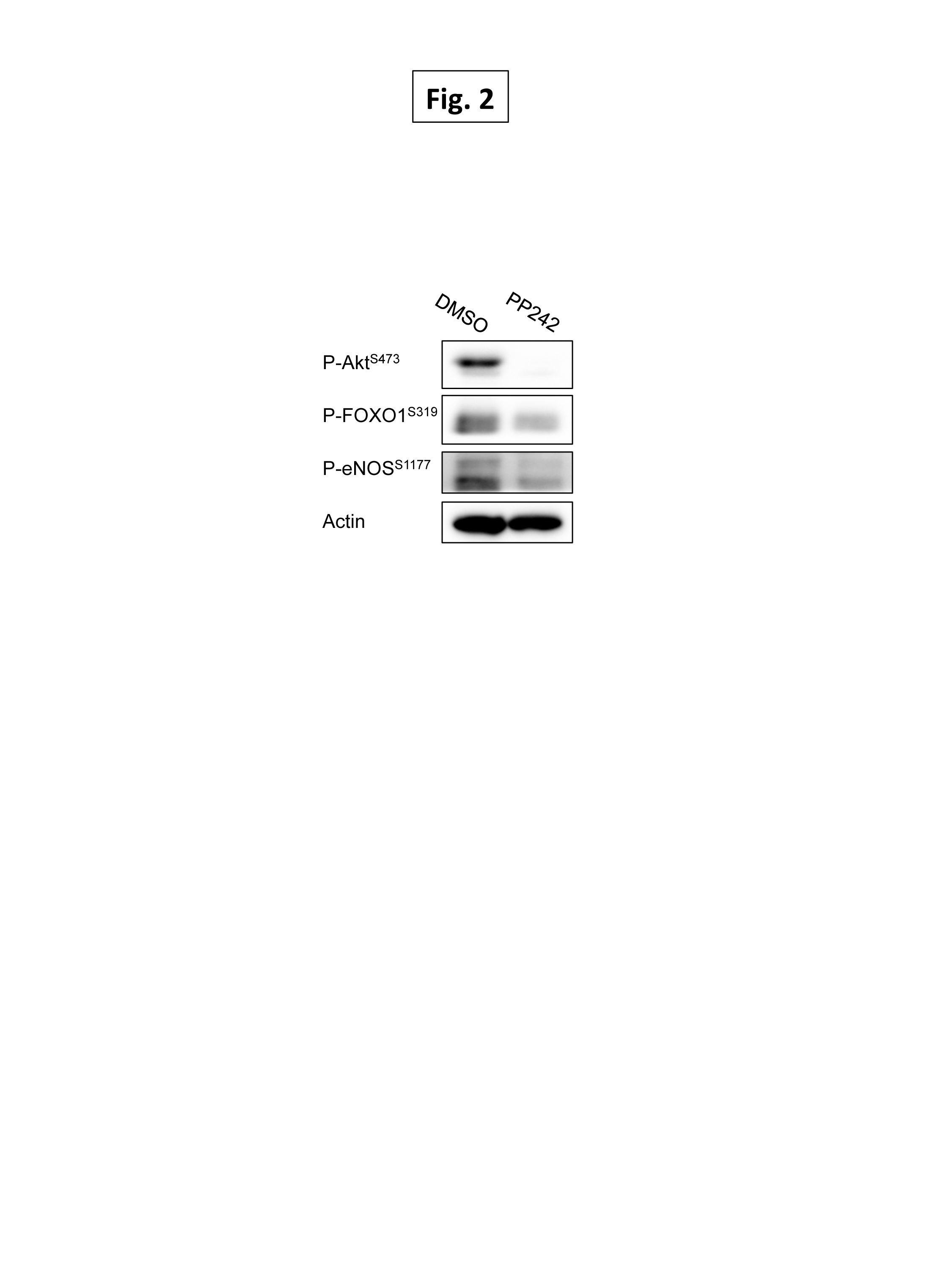

Supplement: S2 Fig — HUVECs were treated with PP242 overnight. A representative Western blot of EC phospho-Akt, phospho-FOXO1/2, phospho-eNOS and actin (n = 3 independent experiments). (TIF) [file pone.0135245.s002.tif]

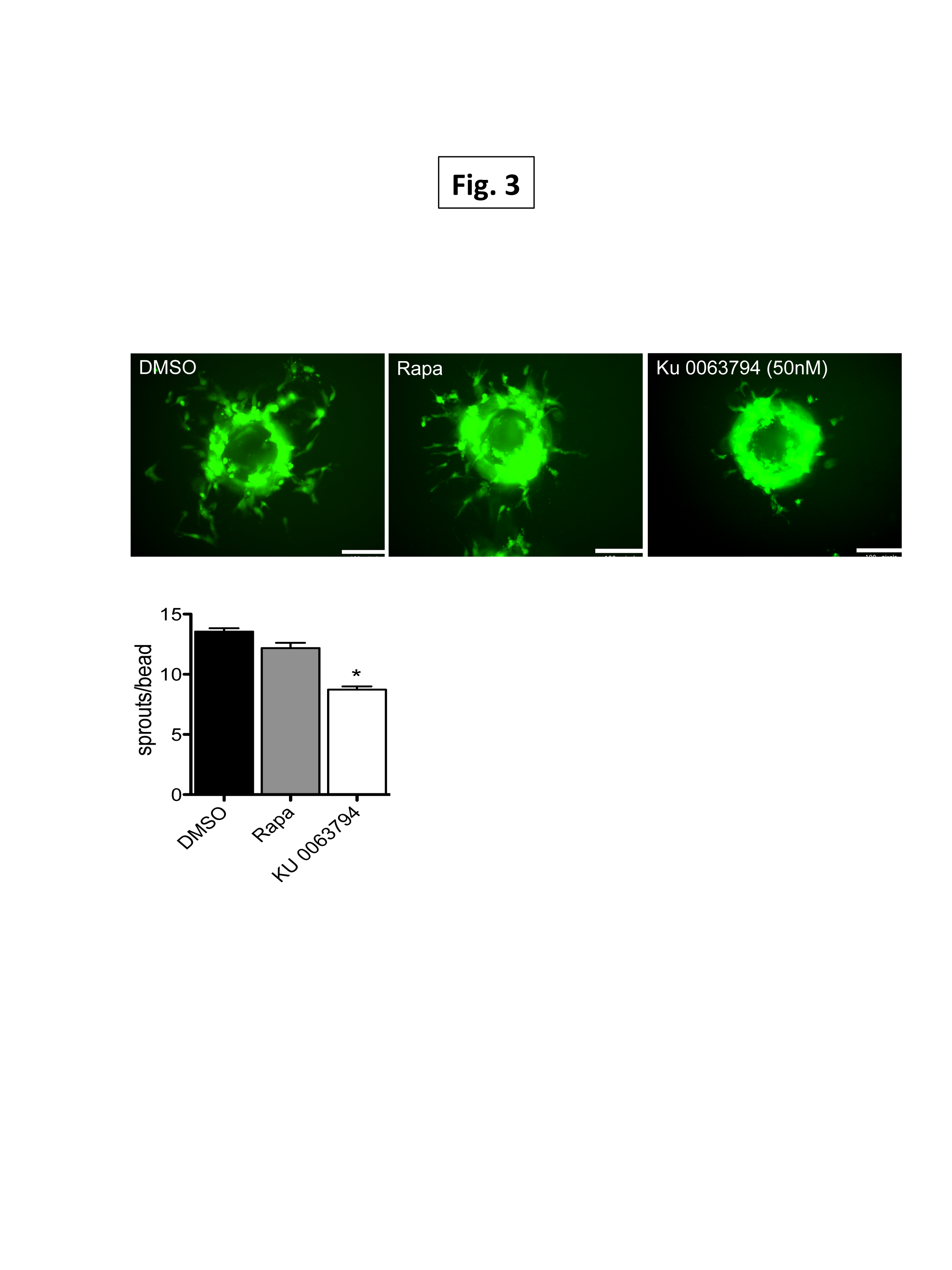

Supplement: S3 Fig — HMEC-1 mounted on Cytodex beads were embedded in a fibrin gel as in Fig 3, were treated by Ku 0063794 (50 nM), Rapamycin, or carrier, and stimulated with VEGF for 18 hours. Representative images of angiogenic sprouting are shown in the upper panels. Quantitation of the number of sprouts per bead (lower panel; n = 4 independent experiments, *P<0.05 by ANOVA, scale bar = 95 um). (TIF) [file pone.0135245.s003.tif]

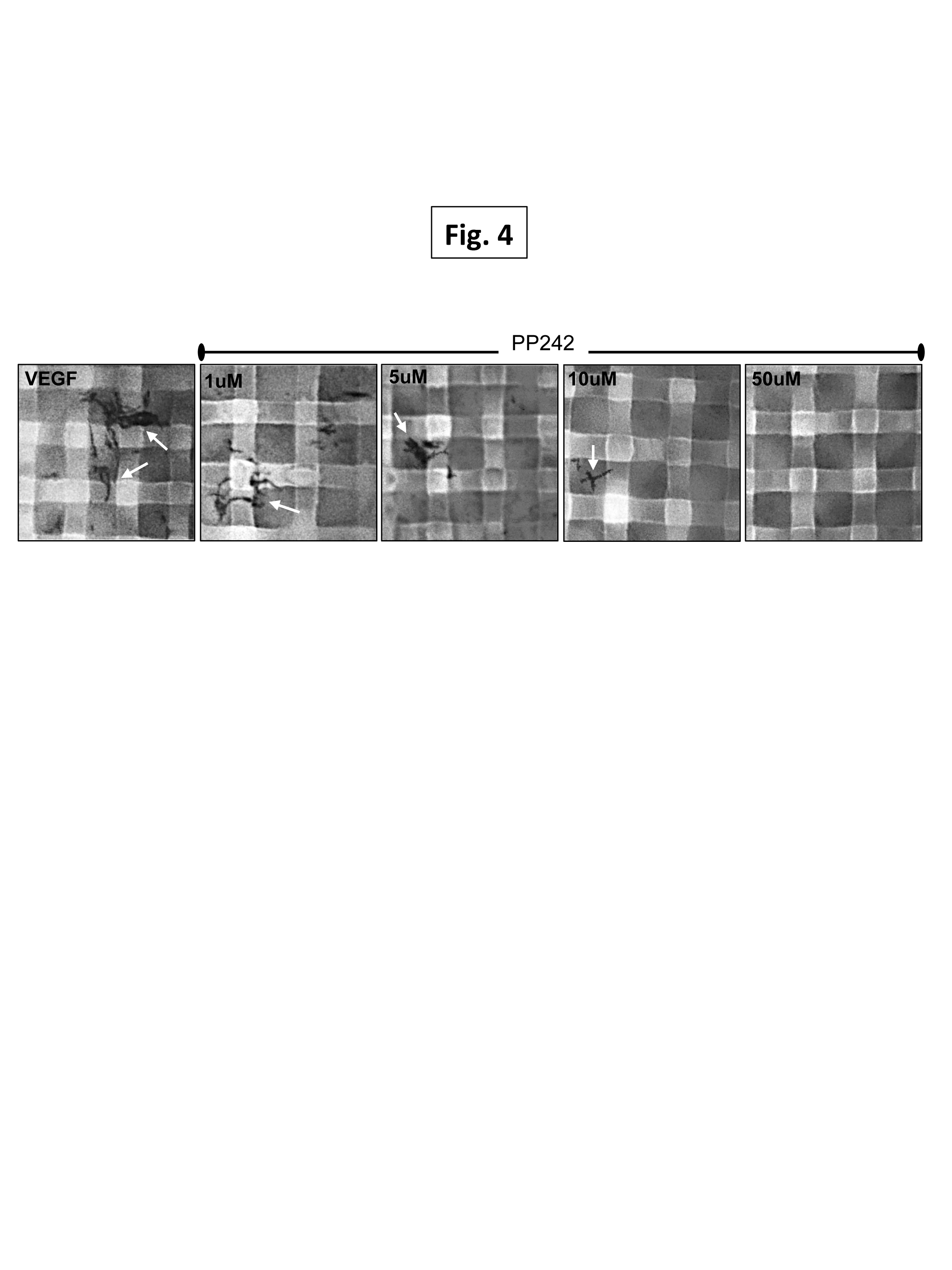

Supplement: S4 Fig — Collagen gels containing VEGF 100 ng/mL, or VEGF + PP242 at the indicated concentration, were cultured on chick CAMs as described in Methods. Newly formed vessels growing into the implant (arrows) are identified. (TIF) [file pone.0135245.s004.tif]

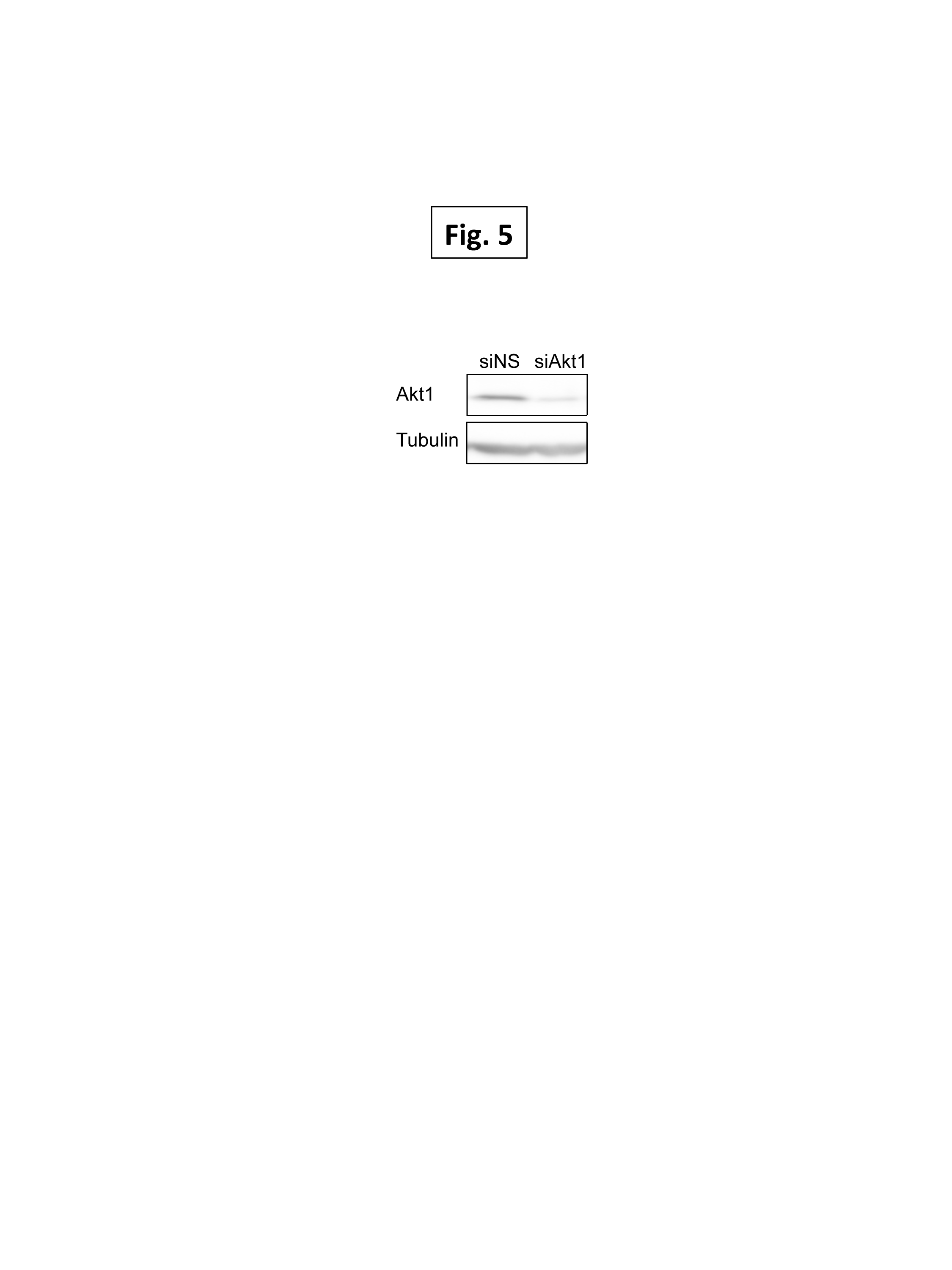

Supplement: S5 Fig — HUVECs were transfected with non-silencing (siNS) or siRNA against Akt1 (siAkt1). Representative Western blot of EC Akt1 and tubulin. (TIF) [file pone.0135245.s005.tif]

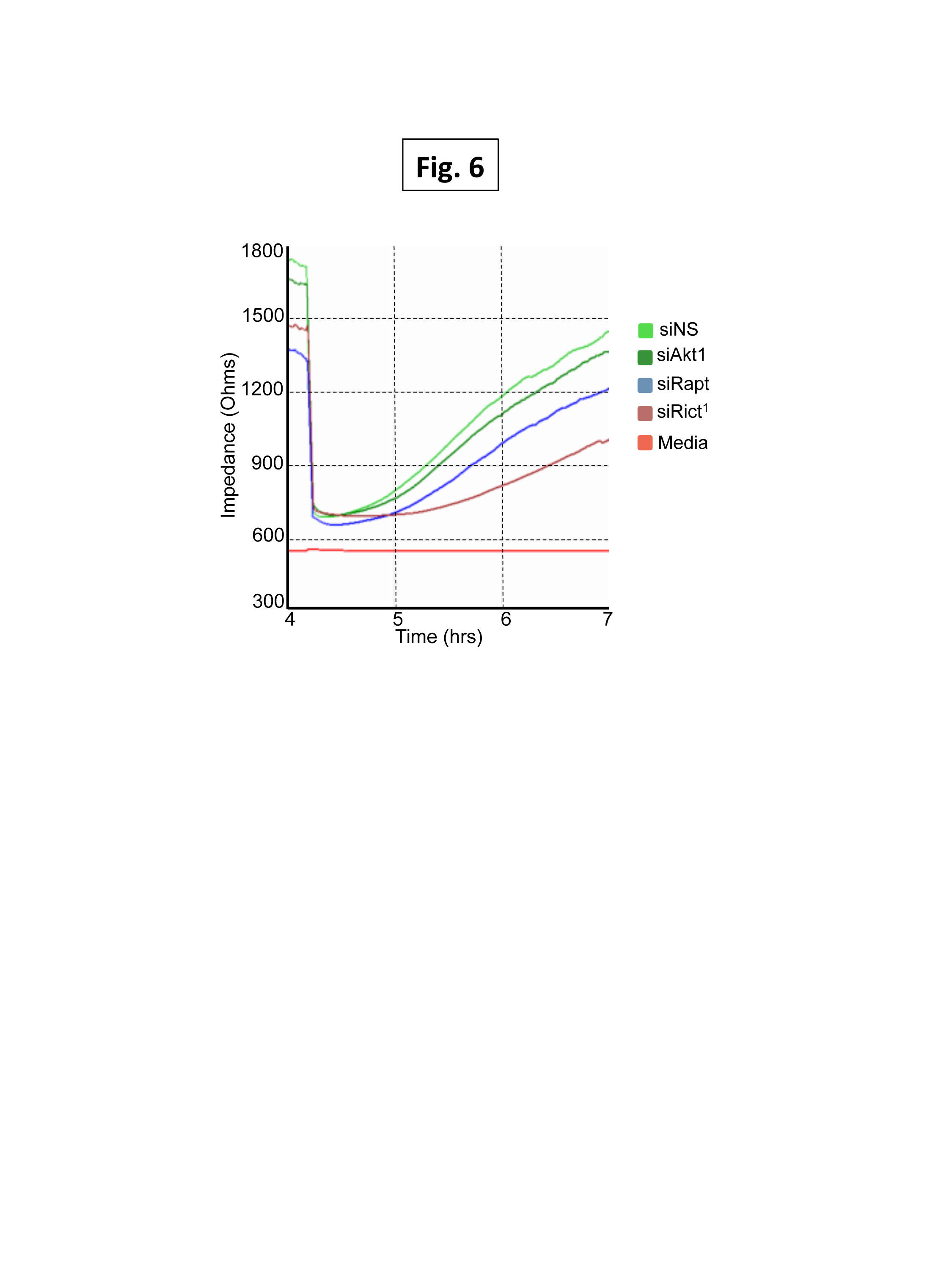

Supplement: S6 Fig — HUVEC were transfected with siRNA against rictor, Akt, or raptor, then seeded on gelatin-coated electrodes at high density and grown to confluence as described in Methods. The monolayer was focally disrupted by an electrical pulse. Continuous electrical impedance values are shown. Representative of 3 independent experiments. (TIF) [file pone.0135245.s006.tif]

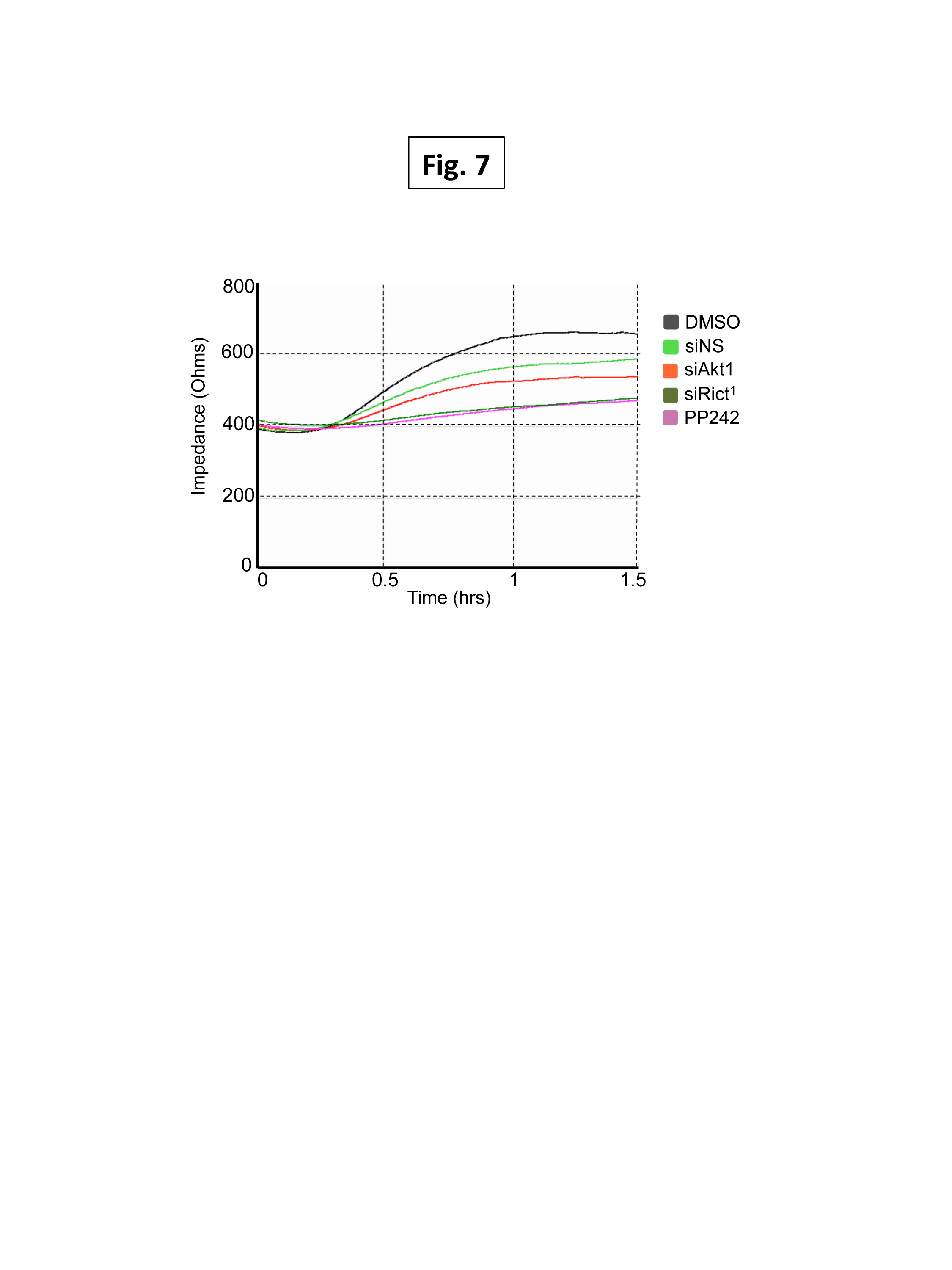

Supplement: S7 Fig — HUVEC were transfected with siRNA against rictor, Akt, or raptor, or treated with PP242 as indicated. The EC were then seeded on gelatin- coated electrodes as described in Methods. Continuous electrical impedance values are shown. Representative of 3 independent experiments. (TIF) [file pone.0135245.s007.tif]
